# Antibiotic resistance and bacterial co-infections in COVID-19 patients in Iran: a systematic review and meta-analysis of hospitalized and non-hospitalized cases

**DOI:** 10.1186/s12879-025-11643-6

**Published:** 2025-09-29

**Authors:** Zahra Najafi-Olya, Zahra Heydarifard, Mehdi Azizmohammad Looha, Akram Sadat Ahmadi, Neda Yarhamadi, Moslem Safaei

**Affiliations:** 1 Department of Virology, Faculty of Medicine, Lorestan University of Medical Sciences, Khorramabad, Iran; 2 Hepatitis Research Center, Lorestan University of Medical Sciences, Khorramabad, Iran; 3 Department of Microbiology, School of Medicine, Lorestan University of Medical Sciences, Khorramabad, Iran; 4 Basic and Molecular Epidemiology of Gastrointestinal Disorders Research Center, Research Institute for Gastroenterology and Liver Diseases, Shahid Beheshti University of Medical Sciences, Tehran, Iran; 5 Department of Virology, School of Public Health, Tehran University of Medical Sciences, Tehran, Iran

**Keywords:** Antibiotic resistance, COVID-19, Iran, Carbapenem resistance, Multi-drug resistance, ICU

## Abstract

**Background:**

The COVID-19 pandemic exacerbated antimicrobial resistance (AMR) in Iran, where up to 100% of hospitalized patients received antibiotics despite low bacterial co-infection rates. However, no comprehensive review has evaluated the burden of bacterial co-infections, antibiotic resistance (AR), and multi-drug resistance (MDR) across both hospitalized and non-hospitalized Iranian COVID-19 patients. This systematic review and meta-analysis aimed to determine the prevalence of bacterial infections, AR, and MDR during the COVID-19 era in Iran.

**Methods:**

Following PRISMA 2020 guidelines, we systematically searched MEDLINE (PubMed), Embase, Scopus, Web of Science, and Iranian regional databases for studies published from January 2020 to March 2025. The review protocol was registered with the Open Science Framework (OSF; 10.17605/OSF.IO/SCBRX). Fifteen studies comprising 36,403 COVID-19 patients—33,989 inpatients (including 22,875 treated in intensive-care units) and 2,414 outpatients—met the inclusion criteria. We combined results from multiple studies to determine overall rates of bacterial infections, antibiotic resistance, and multi-drug resistance, using methods that account for differences between studies.

**Results:**

The pooled prevalence of bacterial co-infection among Iranian COVID-19 patients was 19.6% (95% CI: 17.8–21.4%) across hospitalized and non-hospitalized settings. Among hospitalized patients, secondary bacterial infections were predominantly caused by Gram-negative pathogens: *Klebsiella pneumoniae* (36.2%), *Acinetobacter baumannii* (28.4%), *Escherichia coli* (24.8%), and *Pseudomonas aeruginosa* (11.7%). High heterogeneity (I² >90%) was observed across most pooled estimates. Critical antimicrobial resistance rates were observed particularly in hospitalized settings, with carbapenem resistance reaching 91% for imipenem and 88% for meropenem in A. baumannii. Given that 63% of the cohort were ICU patients, the high secondary bacterial infection and resistance rates likely reflect risk factors such as mechanical ventilation and prolonged hospitalization.

**Conclusion:**

Iran’s AMR crisis during the COVID-19 era affects both hospitalized and non-hospitalized patients, with particularly concerning rates in healthcare settings. The predominance of hospitalized cases in available literature (ICU-heavy cohorts) reflects the urgent need for antimicrobial stewardship programs targeting hospital-based carbapenem-sparing regimens, while highlighting the need for improved surveillance across all COVID-19 care settings.

**Supplementary Information:**

The online version contains supplementary material available at 10.1186/s12879-025-11643-6.

## Introduction

Antibiotics are often empirically prescribed for suspected bacterial infections both at hospital admission and during hospital stays, which increases the risk of antibiotic resistance (AR) [1]. The COVID-19 pandemic has exacerbated concerns about AR due to widespread, often inappropriate, antibiotic use in healthcare institutions and communities [2, 3]. Although bacterial co-infection rates among hospitalized COVID-19 patients are relatively low, ranging from 5 to 27%, broad-spectrum antibiotics have commonly been prescribed for both prophylaxis and treatment [4]. A global analysis revealed that approximately 72% of COVID-19 patients received antimicrobial treatment, while only 8% experienced bacterial or fungal co-infections [1]. The urgency of the pandemic led researchers to empirically repurpose various medications, including some antibiotics, for their potential antiviral effects against SARS-CoV-2. For instance, azithromycin was widely prescribed, often disregarding antimicrobial stewardship principles [1, 2] While the antiviral efficacy of such approaches remains unproven, the increased antibiotic use has likely contributed to rising AR rates [5].

A recent systematic review and meta-analysis estimated the prevalence of bacterial co-infection at only 5.3% (95% CI 3.8–7.4) and secondary bacterial infection at 18.4% (14.0–23.7) in COVID-19 patients. Alarmingly, among those with bacterial infections, 60.8% (95% CI 38.6–79.3) were resistant to antimicrobials, with 37.5% (26.9–49.5) of isolates showing resistance [6].

Since early 2020, this global situation has led to the emergence of highly resistant microorganisms, potentially worsening outcomes for some patients, particularly those in intensive care units (ICUs). Some COVID-19 patients have experienced fatal co-infections with pan-resistant bacteria. *Staphylococcus aureus* and *Acinetobacter baumannii* are among the most resistant to extended-spectrum antibiotics commonly used to treat life-threatening bacterial infections [7]. COVID-19 patients admitted to intensive care units (ICUs), often requiring intubation, face an increased risk of ventilator-associated pneumonia, particularly from Gram-negative bacteria such as *Pseudomonas aeruginosa*, *A. baumannii*, and *Klebsiella pneumoniae*, as well as Gram-positive bacteria like *S. aureus* [8].

The situation in Iran presents a complex picture. While national antibiotic consumption decreased by approximately 30% during the pandemic, primarily due to reduced outpatient visits, hospital prescribing remained extremely high. Studies have shown that up to 100% of hospitalized COVID-19 patients in Iran received antibiotics despite low co-infection rates, highlighting significant antimicrobial stewardship challenges [5]. A recent retrospective study in Iran found a significant increase in antibiotic resistance among Gram-negative bacteria—particularly *P. aeruginosa* and *K. pneumoniae*—during the COVID-19 period compared to the pre-pandemic era [5]. This trend occurred despite changes in hospital protective procedures that may have reduced the overall prevalence and variety of bacteria in healthcare settings.

To date, no comprehensive review has evaluated the burden of bacterial co-infections and antibiotic resistance during COVID-19 in Iran. This study aims to address this knowledge gap by systematically determining the prevalence of bacterial infections, antibiotic resistance (AR), and multi-drug resistance (MDR) during the COVID-19 era in Iran through a comperhensive assessment of the available evidence.

## Materials and methods

### Protocol registration and reporting guidelines

This systematic review was conducted according to a predetermined protocol that was retrospectively registered with the Open Science Framework (OSF) on July 23, 2025 (Registration DOI: 10.17605/OSF.IO/SCBRX). The complete protocol, including detailed methodology, search strategies, and analysis plans, is publicly available through the OSF registration. This systematic review and meta-analysis was reported according to the Preferred Reporting Items for Systematic Reviews and Meta-Analyses (PRISMA) 2020 statement. The completed PRISMA checklist is provided as Supplementary Material.

### Study selection and data management

**Fig. 1:**
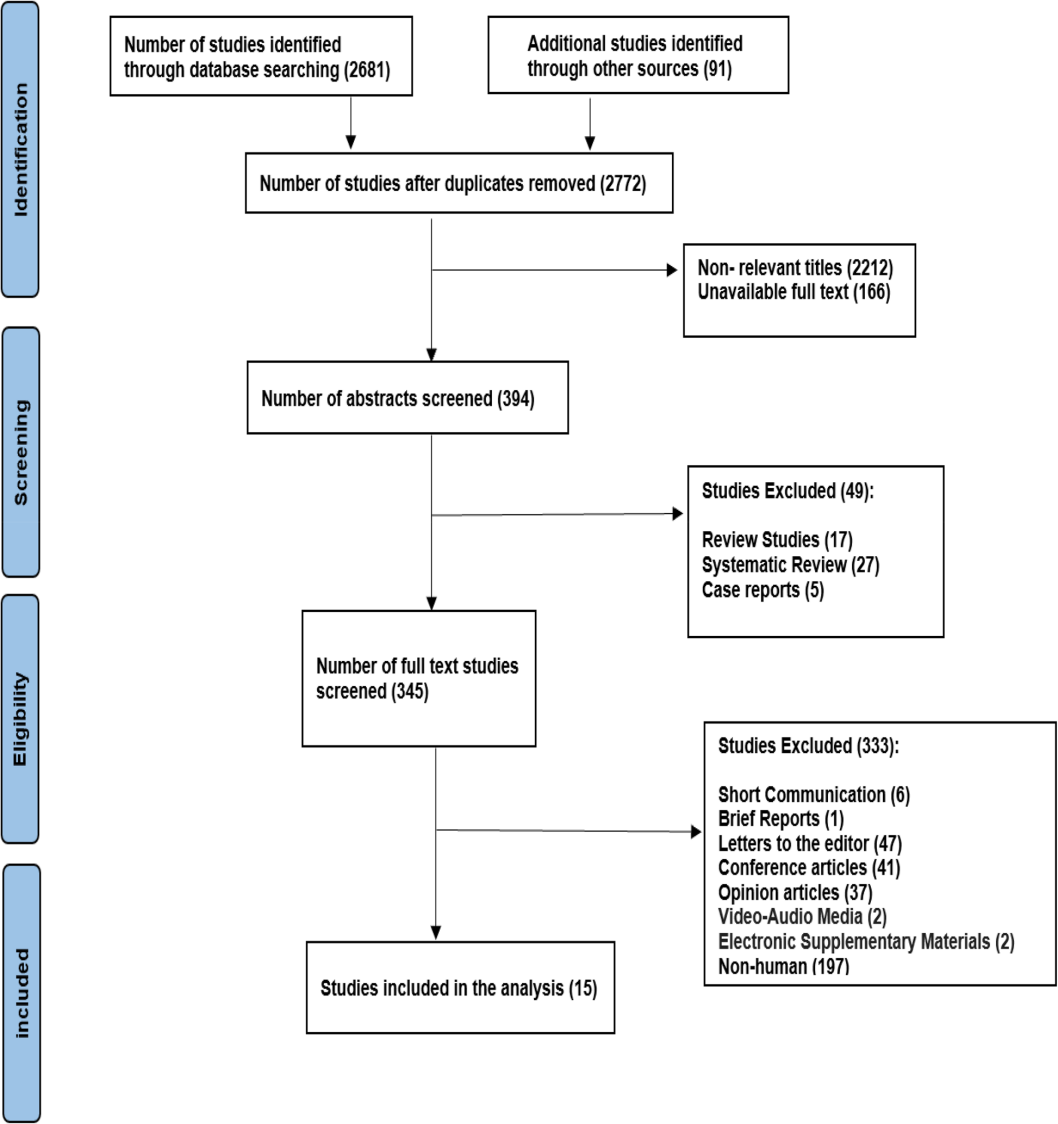
PRISMA 2020 flow diagram for systematic review of antibiotic resistance in Iranian COVID-19 patients

All records obtained through the systematic search strategy were imported into EndNote X9 software for reference management, where duplicate articles were identified and removed using both automated and manual screening processes. The titles and abstracts of identified articles were screened independently by two reviewers (A.S.A. and N.Y.) using predetermined inclusion and exclusion criteria. Disagreements between reviewers were resolved through discussion, and when consensus could not be reached, a third reviewer (Z.H.) was consulted for final decision. Full-text articles of potentially eligible studies were retrieved and assessed for final inclusion. Figure 1 presents the PRISMA flow diagram for study selection.

### Eligibility criteria

In this review, studies were included based on the following conditions: they involved hospitalized patients or a mix of hospitalized and non-hospitalized patients during the COVID-19 era in Iran, excluding studies that solely focused on community patients. The search was limited to English full-text articles published between January 1, 2020, and March 2025. Eligible study designs included original studies, case-control studies, cohort studies, and cross-sectional studies. To be considered, studies were required to report data on antibiotic resistance, COVID-19, and Iran. Exclusion criteria included reviews, opinion articles, letters, short communications without data, studies with missing outcomes of interest (bacterial identification, antibiotic susceptibility data, or resistance patterns), and studies with incomplete methodological information preventing quality assessment. Gray literature was considered only if available for the study. Duplicate articles, case reports, letters to the editor, conference articles, commentaries, systematic reviews, viewpoints, and articles written in languages other than English were also excluded. Studies reporting on AR in non-human populations were omitted. Only studies presenting complete data and meeting the predefined criteria for the outcomes of interest were included in this study.

### Information sources and search strategies

We searched nine bibliographic databases—MEDLINE (PubMed), Embase (Elsevier), Scopus, Web of Science Core Collection, the Cochrane Library (CENTRAL), SciELO, LILACS, Redalyc, and ScienceDirect—for studies published from 1 January 2020 to 31 March 2025. Controlled vocabulary and free-text terms for three concept blocks (“COVID-19/SARS-CoV-2,” “antibiotic OR antimicrobial resistance,” and “Iran”) were combined with AND. Database-specific syntaxes are provided in Supplementary Table S1 (e.g., PubMed and Embase searched on 31 March 2025; Scopus and Web of Science on 30 March 2025).To minimize publication bias, we also screened grey literature by running a structured Google Scholar title search (first 300 hits: allintitle: COVID-19 antibiotic resistance Iran), hand-searched proceedings of the Iranian Congress of Microbiology, and reviewed reports from the Iranian Ministry of Health and the Iranian Registry of Clinical Trials. Automatic and manual de-duplication was performed in EndNote X9 before screening commenced.

### Data extraction and management

Relevant data were extracted and entered into Microsoft Excel, including the name of the first author/year, province, period, study design, sample size, setting, age, specimens, type of bacteria isolated and its number of isolations, type of antibiotic resistance, and quality of included articles (Table 1). Key findings and related parameters from each study were also extracted and entered into the electronic form. Extracted information was compared with the original study and corrected if discrepancies were found, if needed, the differences were resolved by the corresponding author. Throughout this manuscript, the phrase secondary bacterial infection refers to infections identified after a confirmed SARS-CoV-2 diagnosis (hospital or community), whereas bacterial co-infection is reserved for pathogens detected concurrently with the index COVID-19 diagnosis.

**Table 1:** Overview of the studies reporting antibiotics resistance during COVID-19 in Iran

Author, Year	Province	Study Period	Study Design	Participants	Setting	Mean Age (years)	Specimens	Most Common Bacteria	Key Antibiotic Resistance Findings	Quality Score
Khavandegar, 2025[31]	Tehran	March 2017-September 2022	retrospective	14,268 patients	ICU patients	---	Respiratory, blood, urine	*Klebsiella spp*. (26.5%), *Acinetobacter spp.* (19.9%), *E. coli* (12.7%), *Staphylococcus spp*. (12.1%), *P. aeruginosa* (8.8%)	Significant increase in resistance during COVID-19 era compared to pre-COVID era	80% (High)
Ghamari, 2025[15]	Tehran	April-November 2021	Cross-sectional	43 COVID-19 patients with A. baumannii co-infection	ICU patients	66.7 ± 11	Respiratory tract (81.4%), blood (14%), urine (4.6%)	A. baumannii (100%)	97.7% resistance to imipenem, meropenem, piperacillin-tazobactam and ciprofloxacin; 95.3% to cefepime; 93% to gentamicin; 90.7% to trimethoprim-sulfamethoxazole and amikacin; 44.2% to colistin; 91% were XDR phenotype	70% (High)
Abniki, 2024[32]	Isfahan	March 2021-January 2023	Cross-sectional	3,651 clinical samples	inpatients	57.4 ± 8	Urine, blood, respiratory secretions, wounds	*A. baumannii* (46.04%), *K. pneumoniae* (44.94%), *P. aeruginosa* (9.01%)	*A. baumannii*: highest resistance to Ciprofloxacin (98.0%) and Ampicillin-Sulbactam (97.0%); *K. pneumoniae*: highest resistance to Ampicillin-Sulbactam (89.3%) and Ciprofloxacin (83.6%); *P. aeruginosa*: highest resistance to Trimethoprim-Sulfamethoxazole (90.3%) and Levofloxacin (67.5%)	70% (High)
Raoofi, 2023[5]	Fars	2 months pre-pandemic, 12 months pandemic	Retrospective observational	2,675 patients (1,778 pre-pandemic, 897 pandemic)	625 inpatients, 2,050 outpatients	60 ± 22	Urine, sputum, blood	*E. coli* (30%), *P. aeruginosa* (10%), *Staphylococcus strains* (4%), *K. pneumoniae* (3.4%)	General rise in resistance among Gram-negative bacteria (excluding E. coli); resistance to gentamicin, cefepime, and cefotaxime increased overall	60% (Medium)
Salarvand, 2023[33]	Tehran	Pandemic period	Cross-sectional	1,755 severe patients	ICU patients	Not reported	Peripheral blood	*K. pneumoniae* (22.1%), *S. epidermidis* (7.9%), *coagulase-negative Staphylococcus* (15.0%)	Resistance prevalence: 85.9%; highest for cotrimoxazole (61.7%), ciprofloxacin (51.3%), imipenem (50.0%); MDR rate: 96.3%	50% (Medium)
Mobarak Qamsari, 2023[18]	Tehran	March 2021-July 2022	Cross-sectional	101 COVID-19 patients	ICU patients	61 ± 12.45	ETT, BAL, sputum	*K. pneumoniae* (41.5%), *A. baumannii* (20.8%), *S. aureus* (4.9%)	Significant increase in resistance: *K. pneumonia*e (88% to cefazolin, 68% to levofloxacin); A. baumannii (100% to meropenem, 96% to multiple antibiotics); S. aureus (100% to penicillin)	70% (High)
Besharati, 2023[34]	North Khorasan	Pre-pandemic and pandemic	Cross-sectional	752 (376 pre-COVID, 376 post-COVID)	Healthcare workers	Not reported	Nasal swab	Pre-COVID: *S. aureus* (27.9%), *S. epidermidis* (12%); Post-COVID: *S. aureus* (21%), *S. epidermidis* (21%)	Increased resistance in post-COVID period; highest against penicillin, lowest against vancomycin	60% (Medium)
Mohammadnejad, 2023[35]	Tehran	March 2020-January 2022	Retrospective cross-sectional	6,524 COVID-19 patients	ICU patients	52 ± 14.4	Respiratory	*K. pneumoniae* (31.6%), *E. coli* (15.8%), *A. baumannii* (15.7%)	VRE prevalence: 88%; KPC prevalence: 82%; 7.3% of A. baumannii resistant to colistin	60% (Medium)
Shiralizadeh, 2023[36]	Hamadan	December 2020-July 2021	Cross-sectional	15 COVID-19 patients	ICU patients	15–105 years (range)	ETT	*P. aeruginosa*	Highest resistance to imipenem (93.3%), trimethoprim-sulfamethoxazole (93.3%), ceftriaxone (80%), ceftazidime (80%)	60% (Medium)
Nikzad Jamnani, 2022[37]	Mazandaran	February-September 2020	Retrospective	40 patients (22 COVID-19, 18 non-COVID-19)	ICU patients	40–70 years (range)	ETT	COVID-19 group: *A. baumannii* (41%), *P. aeruginosa* (9.1%); Non-COVID 19 group: *A. baumannii* (22.2%), *K. pneumoniae* (11.1%)	A. baumannii: 100% resistance to multiple antibiotics; K. pneumonia: 100% resistance to multiple antibiotics; both susceptible only to colistin in non-COVID group	70% (High)
Akrami, 2022[38]	Ahvaz	January-April 2021	Cross-sectional	77 severe COVID-19 patients	ICU patients	61 ± 14.44 (32–96 years)	ETT secretion	*K. pneumoniae* (28.4%), *S. aureus* (22.4%), *H. influenzae* (10.4%, PCR), *M. pneumoniae* (11.7%, PCR)	> 70% resistance to most antibiotics including fluoroquinolones, carbapenems, and cephalosporins; 68.7% MDR isolates	80% (High)
Moradi, 2021[39]	Zanjan	February 2019-February 2020	Cross-sectional	5,530 COVID-19 cases	5,166 inpatients, 364 outpatients	Not reported	Respiratory, blood, urine	Urine: *E. coli* (12.5%); Respiratory: *A. baumannii* (15.4%); Blood: *A. baumannii* (2.1%)	Gram-negative: 91.7% resistance to cefixime, 89.64% to trimethoprim/sulfamethoxazole; Gram-positive: 90.91% to ciprofloxacin, 83.33% to tetracycline	70% (High)
Khoshbakht, 2022[40]	Mashhad	January 2020-January 2022	Cross-sectional	1,672 clinical samples	Inpatients	54.77 ± 24.16	Urine, blood, respiratory	*E. coli* (36.8%), *P. aeruginosa* (21.6%), *K. pneumoniae* (20.9%), *A. baumannii* (20.5%)	E. coli: 89.6% resistance to ampicillin; *K. pneumoniae*: 98% to ampicillin; P. aeruginosa: 91.8% to imipenem; A. baumannii: 94.6% to ceftazidime	70% (High)
Hesari, 2022[41]	Bojnourd	April 2019-March 2021	Cross-sectional	4,560 clinical samples	Inpatients and outpatients	0–91 years (range)	Urine	*E. coli* (66.66%), *K. pneumoniae* (19.84%), *Enterobacter* (4.8%)	High resistance to 3rd and 4th generation cephalosporins; ciprofloxacin most effective (15.38% resistance)	50% (Medium)
Ghanizadeh, 2021[42]	Kashan	May-September 2020	Cross-sectional	70 COVID-19 patients	ICU patients	66.16 ± 10.52	ETT	*K. pneumoniae* (77.14%)	Resistance rates: meropenem (80.4%), cefepime/aztreonam/piperacillin-tazobactam (70%), tobramycin (61.4%), ciprofloxacin (57.7%), gentamicin (55.7%), imipenem (50%)	70% (High)

Abbreviations *ETT* Endotracheal tube, *BAL* Bronchoalveolar lavage, *ICU* Intensive care unit, *MDR* Multi-drug resistant, *VRE* Vancomycin-resistant enterococci, *KPC* Klebsiella pneumoniae carbapenemase, *XDR* Extensively drug-resistant

### Quality assessment

The Joanna Briggs Institute’s critical appraisal tool was employed to evaluate the quality of the included studies (https://jbi.global/critical-appraisal-tools). This tool consists of 8 items for assessing cross-sectional studies and 11 items for case-control studies. Each cross-sectional study received a score between 0 and 8, while case-control studies were scored from 0 to 11. Consequently, the quality of the studies was ranked as high (for a score ≥ 70%), medium (for scores between 50% and 69%), and low (for scores < 50%). All authors participated in the assessment process, and any issues that arose during the evaluation were resolved through discussion among the authors.

### Outcome measures

The primary objective of this review was to determine the pooled prevalence of bacterial infections in hospitalized and non-hospitalized patients during the COVID-19 era in Iran. Additionally, this review aimed to investigate AR resulting from antibiotic use and MDR during the COVID-19 era. All suspected COVID-19 patients were included, regardless of SARS-CoV-2 test confirmation. The study then pooled the prevalence of isolated bacteria and antibiotic-resistant bacteria. Bacterial infection prevalence was defined as the proportion of patients with at least one positive culture among the total number of patients studied. Bacteria considered in this review included *Acinetobacter baumannii*, *Escherichia coli*,* Klebsiella pneumoniae*,* Pseudomonas aeruginosa*,* Staphylococcus aureus* and *Staphylococcus coagulase negative*.

### Data synthesis

Meta-analyses were performed to quantitatively synthesize the prevalence of antibiotic resistance across different bacterial species, as well as the pooled prevalence of MDR. All analyses were conducted in R (version 4.2.3) using the meta and metafor packages.

For the proportion meta-analyses, the number of resistant isolates (events) and the total number tested (sample size) were extracted for each included study. The Freeman–Tukey double arcsine transformation (PFT) was applied using the metaprop function to stabilize the variance in proportions close to 0 or 1. Analyses were conducted under a random-effects model with the DerSimonian–Laird estimator to account for between-study heterogeneity. In parallel, logit-transformed event rates were computed using escalc (measure = “PLO”) in the metafor package and pooled using the restricted maximum likelihood (REML) method via rma.

For studies reporting MDR rates (defined as resistance to at least two or more antibiotic classes), pooled MDR proportions were calculated for each bacterial species separately after reshaping the dataset to long format. Proportions were assumed to be based on binomial distributions, with sample sizes normalized to 100 when only percentage values were reported. Subgroup analyses were performed by bacterial species to evaluate differences in MDR rates.

Statistical heterogeneity was assessed using the Q statistic, τ², and the I² index, with I² values above 50% indicating moderate to high heterogeneity. Publication bias was evaluated using funnel plots, Egger’s regression test, and Begg’s rank correlation test. Galbraith (radial) plots were generated to visualize the dispersion of effect sizes. Where appropriate, the trim-and-fill method was applied to correct for potential publication bias and estimate adjusted pooled proportions.

Sensitivity analyses were conducted using a leave-one-out approach by sequentially omitting one study at a time and recalculating the pooled estimate to assess the robustness of the results.

Forest plots, funnel plots, and Galbraith plots were exported in high-resolution JPEG format with RevMan5-style layout and serif fonts to enhance readability. Statistical significance was defined as a two-sided p-value < 0.05.

## Results

### Study selection

A total of 2,772 studies were identified through database searches and secondary citations. After removing duplicates, 2,212 titles and abstracts were screened for relevance. Subsequently, 394 full-text articles were retrieved and assessed for eligibility based on the predefined inclusion and exclusion criteria. Ultimately, 15 studies meeting all criteria were included in this review (Fig. 1).

### Study characteristics

The included studies were published between January 2020 and March 2025 and originated from various provinces in Iran, including Tehran (5 studies), Fars (1 study), Ahvaz (1 study), Mazandaran (1 study), Zanjan (1 study), Mashhad (1 study), Hamadan (1 study), Bojnourd (1 study), North Khorasan (1 study), Kashan (1 study) and Isfahan (1 study). Of these, 12 studies were cross-sectional, one was a cohort study, and two were retrospective observational study. Of the 15 included studies, 9 (60.0%) were rated high quality, 6 (40.0%) medium quality, and none were low quality based on the Joanna Briggs Institute criteria.

Eight studies focused on COVID-19 patients in ICUs, three studies included both ICU and non-ICU wards, and four studies examined inpatient and outpatient populations. In total, the reviewed studies analyzed data from 22,875 ICU patients, 11,114 inpatients, and 2,414 outpatients. Most specimens used in these studies were derived from blood, respiratory secretions, or urine samples (Table 1).

### Prevalence of isolated bacteria

The prevalence of bacteria isolated in hospitalized and non- hospitalized during COVID-19 era in Iran is shown in Table 2. The highest prevalence was *K. pneumoniae* (36.2%; 95% CI [30.5–41.9]), followed by *A. baumannii* (28.4%; 95% CI [22.1–34.7]). *E. coli* showed a prevalence of 24.8% (95% CI [18.3–31.3]), while *P. aeruginosa* was found in 11.7% of cases (95% CI [7.4–16.0]). Among Gram-positive bacteria, *S. aureus* (9.1%; 95% CI [4.2–14.0]) and *coagulase-negative Staphylococcus* (7.5%; 95% CI [2.8–12.2]) were less frequently isolated. The overall pooled prevalence from the six isolated bacteria was 19.6% (95% CI [17.8–21.4]). The heterogeneities for these analyses were high (I² >89%), with particularly high heterogeneity observed for Gram-negative bacteria (I² >96%). The random effects model was used to account for the significant differences between studies.

**Table 2:** The prevalence of bacterial isolated in the hospitalized and non-hospitalized patients during COVID-19 in Iran

Bacterium	Number of Studies Reporting	Number of Isolations	Prevalence (95% CI)	I² (%)	*p*-value
*Acinetobacter baumannii*	7	1,682	28.4% (22.1–34.7)	98.5	< 0.001
*Klebsiella pneumoniae*	8	3,872	36.2% (30.5–41.9)	99.2	< 0.001
*Escherichia coli*	6	1,611	24.8% (18.3–31.3)	97.9	< 0.001
*Pseudomonas aeruginosa*	5	592	11.7% (7.4–16.0)	96.8	< 0.001
*Staphylococcus aureus*	3	315	9.1% (4.2–14.0)	94.3	< 0.001
*Coagulase-negative Staphylococcus*	3	350	7.5% (2.8–12.2)	89.7	< 0.001

Notably, *K. pneumoniae* had the highest number of isolations (3,872) across the included studies, while *A. baumannii* (1,682 isolations) showed a concerning trend of increasing prevalence during the pandemic period, particularly in ICU settings.

### Prevalence of AR and MDR

The systematic analysis of AR during COVID-19 era in Iran revealed a pooled prevalence of 71.8% (95% CI:63.2–79.1%), with substantial heterogeneity (I² >98%) reflecting regional and institutional variability in resistance patterns. Resistance to β-lactams, fluoroquinolones, and carbapenems dominated across Gram-negative pathogens, while Gram-positive organisms exhibited high resistance to aminoglycosides and macrolides (Table 3, for more details, see supplementary data).

**Table 3:** The prevalence of AR and MDR in hospitalized and non-hospitalized patients during COVID-19 in Iran

outcome	Studies (*n*)	Patients (*n*/*N*%)	Prevalence (95% CI)	Heterogeneity I² (95% CI)	*P*-value*
*Staphylococcus aureus*	---	---	---	---	---
*Multidrug resistance*					
*Gentamicin resistance*	2	10(43)	0.42 (0.18, 0.7)	38.44%	0.59
*Ampicillin resistance*	2	9(11)	0.28 (0.01, 0.95)	69.05%	0.633
*Ciprofloxacin resistance*	2	23(74)	0.71 (0.29, 0.94)	69.26%	0.328
*Clindamycin resistance*	2	23(91)	0.88 (0.44, 0.98)	49.92%	0.081
*E. coli*	---	---	---	---	
Multidrug resistance					
Amikacin resistance	3	689(8)	0.32 (0.03, 0.9)	98.32%	0.621
Cefazolin resistance	2	683(46)	0.1 (0, 0.93)	91.77%	0.364
Ciprofloxacin resistance	4	995(30)	0.5 (0.07, 0.93)	99.03%	0.997
Ampicillin resistance	2	683(15)	0.26 (0.04, 0.73)	98.24%	0.313
Cefepime resistance	4	995(18)	0.4 (0.1, 0.8)	98.67%	0.667
Nitrofurantoin resistance	2	683(15)	0.15 (0.13, 0.18)	0%	< 0.001
Trimethoprim/Sulfamethoxazole					
resistance	2	307(42)	0.42 (0.37, 0.48)	0%	0.005
Cefixime resistance	2	69(87)	0.86 (0.76, 0.93)	0%	< 0.001
Cefotaxime resistance	2	312(38)	0.66 (0.09, 0.97)	77.24%	0.653
Cefoxitin resistance	2	621(5.8)	0.4 (0, 0.99)	92.86%	0.886
Ceftazidime resistance	3	689(13)	0.5 (0.04, 0.96)	98.59%	0.991
Ceftriaxone resistance	3	989(70)	0.73 (0.62,0.81)	86.83%	< 0.001
Gentamicin resistance	4	995(35)	0.52 (0.18, 0.85)	98.64%	0.907
Imipenem resistance	3	689(37)	0.37 (0.33, 0.4)	0%	< 0.001
Meropenem resistance	3	689(28)	0.46 (0.19, 0.75)	93.88%	0.786
*K. pneumoniae*					
*Multidrug resistance*	4	---	0.82 (0.64, 0.92)	92.22%	0.002
*Amikacin resistance*	5	2093(60)	0.62 (0.29, 0.86)	98.17%	0.495
*Cefazolin resistance*	2	432(56)	0.91 (0.05, 1)	92.68%	0.386
*Ciprofloxacin resistance*	7	2168(70)	0.67 (0.32, 0.9)	97.62%	0.355
*Ampicillin resistance*	2	432(42)	0.87 (0.02, 1)	94.37%	0.52
*Cefepime resistance*	6	2167(72)	0.69 (0.42, 0.87)	98.28%	0.159
*Nitrofurantoin resistance*	2	432(32)	0.4 (0.16, 0.71)	96.12%	0.549
*Trimethoprim/Sulfamethoxazole*					
*resistance*	2	45(31)	0.37 (0.12, 0.71)	28.3%	0.469
*Cefixime resistance*	2	82(88)	0.87 (0.78, 0.93)	0%	< 0.001
*Cefotaxime resistance*	3	414(11)	0.34 (0.02, 0.91)	97.9%	0.672
*Cefoxitin resistance*	2	370(26)	0.54 (0.06, 0.95)	95.08%	0.911
*Ceftazidime resistance*	5	2093(70)	0.74 (0.37, 0.93)	98.67%	0.196
*Ceftriaxone resistance*	3	476(72)	0.72 (0.68, 0.76)	0%	< 0.001
*Gentamicin resistance*	6	527(44)	0.66 (0.4, 0.85)	93.03%	0.223
*Imipenem resistance*	4	482(66)	0.74 (0.53, 0.88)	88.66%	0.027
*Meropenem resistance*	5	2123(67)	0.73 (0.6, 0.82)	92.73%	0.001
*P. aeruginosa* Multidrug resistance					
Multidrug resistance	2	---	0.59 (0.41, 0.74)	83.01%	0.322
*Amikacin resistance*	4	718(55)	0.7 (0.36, 0.9)	97.57%	0.243
*Cefepime resistance*	6	847(57)	0.6 (0.38, 0.79)	95.62%	0.378
*Cefixime resistance*	2	24(100)	0.94 (0.66, 0.99)	7.94%	0.011
*Ceftazidime resistance*	5	733(62)	0.71 (0.51, 0.85)	92.4%	0.038
*Ciprofloxacin resistance*	7	849(31)	0.48 (0.2, 0.76)	96.74%	0.879
*Colistin resistance*	2	37(5.5)	0.08 (0.01, 0.33)	29.98%	0.006
*Gentamicin resistance*	5	518(60)	0.6 (0.56, 0.64)	0.01%	< 0.001
*Imipenem resistance*	4	404(81)	0.83 (0.73, 0.9)	18.13%	< 0.001
Levofloxacin resistance	3	707(66)	0.67 (0.63, 0.7)	0%	< 0.001
*Meropenem resistance*	4	718(74)	0.79 (0.57, 0.91)	93.53%	0.011
*A. baumannii*					
*Multidrug resistance*	3	---	0.93 (0.07, 1)	94.6%	0.319
*Amikacin resistance*	6	2160(83)	0.88 (0.64, 0.97)	98.17%	0.006
*Cefepime resistance*	5	2151(88)	0.91 (0.63, 0.98)	98.55%	0.012
*Cefixime resistance*	2	91(100)	0.98 (0.87, 1)	11.87%	< 0.001
*Cefotaxime resistance*	3	388(71)	0.87 (0.4, 0.98)	70.46%	0.106
*Ceftazidime resistance*	5	2151(90)	0.9 (0.7, 0.97)	97.35%	0.001
*Ceftriaxone resistance*	2	426(74)	0.84 (0.5, 0.96)	93.21%	0.051
*Ciprofloxacin resistance*	5	2151(82)	0.82 (0.14, 0.99)	97.46%	0.37
*Gentamicin resistance*	5	479(56)	0.87 (0.56, 0.97)	88.19%	0.026
*Imipenem resistance*	5	479(70)	0.91 (0.67, 0.98)	80.78%	0.004
*Meropenem resistance*	5	2151(90)	0.88 (0.72, 0.95)	96.11%	< 0.001
*Nitrofurantoin resistance*	2	91(77)	0.81 (0.49, 0.95)	38.5%	0.056
*Staphylococcus coagulase-negative*	---	---	---	---	---
*Multidrug resistance*					
*Ciprofloxacin resistance*	2	16(94)	0.87 (0.34, 0.99)	47.24%	0.146
*Clindamycin resistance*	2	16(94)	0.87 (0.34, 0.99)	47.24%	0.146
*Gentamicin resistance*	2	16(69)	0.63 (0.22, 0.91)	46.28%	0.554

*P-value denotes the effect size estimate for each antimicrobial resistance outcome

Carbapenem resistance was most pronounced in *A. baumannii* (imipenem: 91% [67–98%], I² = 80.78%; meropenem: 88% [72–95%], I² = 96.11%) and *K. pneumoniae* (imipenem: 74% [53–88%], I² = 88.66%; meropenem: 73% [60–82%], I² = 92.73%). *P. aeruginosa* followed with 83% [73–90%] imipenem resistance (I² = 18.13%) and 79% [57–91%] meropenem resistance (I² = 93.53%), underscoring the critical threat of carbapenemase-producing organisms in critical care settings.

Resistance to third-generation cephalosporins was widespread, particularly in *K.pneumoniae* (ceftazidime: 74% [37–93%], I² = 98.67%) and *E. coli* (ceftriaxone: 73% [62–81%], I² = 86.83%). Fluoroquinolone resistance exceeded 70% in *S. aureus* (ciprofloxacin: 71% [29–94%], I² = 69.26%) and *A.baumannii* (82% [14–99%], I² = 97.46%), with *P. aeruginosa* demonstrating moderate resistance (48% [20–76%], I² = 96.74%).

*Coagulase-negative staphylococci* exhibited high ciprofloxacin resistance (87% [34–99%], I² = 47.24%), while *S. aureus* showed concerning clindamycin resistance (88% [44–98%], I² = 69.05%). Vancomycin resistance remained rare overall (1.26% [0.46–2.05%]) but reached 88% [83–93%] in one ICU study, highlighting localized outbreaks.

Northern Iran reported elevated resistance in *E. coli* (ampicillin: 88% [44–98%], I² = 49.92%) and *K. pneumoniae* (cefazolin: 91% [5–100%], I² = 92.68%), while Central Iran documented extreme carbapenem resistance in *P. aeruginosa* (amikacin: 70% [36–90%], I² = 97.57%). ICU-specific data revealed higher resistance burdens, particularly for *A. baumannii* (ceftazidime: 90% [70–97%], I² = 97.35%) and *K. pneumoniae* (nitrofurantoin: 40% [16–71%], I² = 96.12%).

The pooled prevalence of MDR pathogens among COVID-19 patients in Iran was 73.4% (95% CI: 64.0–82.8%), derived from a meta-analysis of three high-burden bacterial species, with extreme heterogeneity across studies (I² >90%). MDR *K. pneumoniae* demonstrated the highest prevalence at 0.82 (95% CI: 0.64–0.92; I² = 92%), followed by MDR *A. baumannii* at 0.93 (95% CI: 0.07–1.00; I² = 94.6%), and MDR *P. aeruginosa* at 0.59 (95% CI: 0.41–0.74; I² = 83%). These estimates reflect weighted averages accounting for study-specific variances, though the wide confidence intervals—particularly for *A. baumannii* indicate substantial uncertainty in regional resistance patterns. Insufficient data precluded analysis of MDR prevalence in other bacterial species, including *E.coli* and *Enterococcus spp*.

### Publication bias

Although some asymmetry was visually observed in the funnel plots, indicating potential small-study effects, Egger’s regression test did not detect statistically significant publication bias (*p* > 0.05).

## Discussion

In this systematic review and meta-analysis of 15 studies comprising 36,403 patients (33,989 inpatients and 2,414 outpatients) during the COVID-19 era in Iran, we found a pooled bacterial co-infection prevalence of 19.6% (95% CI: 17.8–21.4%). This prevalence is notably higher than those reported in several large-scale global meta-analyses conducted during the early and middle phases of the pandemic. For example, Langford et al. (2020) reported a pooled prevalence of 6.9% (95% CI: 4.3–9.5%) from 24 studies [9], while Lansbury et al. (2020) found a prevalence of 7% (95% CI: 3–12%) among hospitalized COVID-19 patients across 30 studies [10]. More recently, a comprehensive meta-analysis of 148 studies published in 2023 estimated the global bacterial co-infection prevalence in COVID-19 patients at just 5.3% (95% CI: 3.8–7.4%), with secondary bacterial infections at 18.4% (95% CI: 14.0–23.7%) [6]. However, our findings are more closely aligned with some other meta-analyses that reported higher co-infection rates. For instance, a meta-analysis of 49 studies worldwide documented a bacterial co-infection prevalence of 26.84% (95% CI: 23.85–29.83%) in hospitalized COVID-19 patients [11]. Similarly, Musuuza et al. (2021), pooling data from 118 studies, reported a bacterial co-infection prevalence of 19% (95% CI: 14–25%) and a superinfection prevalence of 24% (95% CI: 19–30%) [12]. In addition, a global meta-analysis found an even higher pooled prevalence of bacterial co-infection at 20.97% (95% CI: 15.95–26.46%) [13]. Several factors may explain both the similarities and differences between our findings and those of previous meta-analyses. First, our study included both inpatients and outpatients, whereas many global reviews focused exclusively on hospitalized or ICU patients. Although outpatients represented a smaller proportion of our cohort, their inclusion provides a broader epidemiological picture and may have contributed to a higher overall prevalence compared to studies with more restrictive inclusion criteria. Second, the temporal scope of our review extended through March 2025, capturing data from the later stages of the pandemic and post-pandemic period. This broader timeframe likely reflects the cumulative impact of prolonged antibiotic pressure, evolving hospital infection control practices, and the emergence of highly resistant bacterial strains in Iran, such as carbapenem-resistant *A. baumannii* and *K. pneumoniae* [14, 15]. It is important to acknowledge that differences in study populations, surveillance techniques, definitions of resistance, and healthcare environments likely contributed to the observed heterogeneity. Therefore, the aggregated estimates should be interpreted as indicative trends rather than exact figures. Efforts to standardize surveillance methodologies across countries are crucial to enable more accurate evaluations [14, 15].

Bacterial co-infection rates were significantly elevated in ICU COVID-19 patients compared to those in general wards. Our meta-analysis included a substantial proportion of ICU patients (63%; 22,875 of 36,403 patients), which likely contributed to our higher overall bacterial co-infection prevalence of 19.6% (95% CI 17.8–21.4%). ICU environments present increased risks for nosocomial infections due to invasive mechanical ventilation, central venous catheterization, and prolonged hospitalization periods, coupled with COVID-19-associated immunocompromise [16, 17]. Sharifipour et al. [7]. identified substantial bacterial co-infections in COVID-19 patients admitted to ICUs in Iran, while Ippolito et al. [8]. documented that ventilator-associated pneumonia—particularly common in COVID-19 ICU patients—significantly increased the risk of resistant Gram-negative infections. Ghamari et al. [15]. further reported that critically ill COVID-19 patients in Tehran ICUs experienced significantly higher rates of *A. baumannii* infections with extensive drug resistance patterns, and Mobarak-Qamsari et al. observed that respiratory tract superinfections with *K. pneumoniae*,* A. baumannii*,* and S. aureus* were substantially more common in ventilated COVID-19 patients, with secondary infection rates approaching 36.2% for *K. pneumoniae* [18]. This ICU-predominant cohort explains the notable difference between our findings and the lower global average.

Our meta-analysis revealed a distinct bacterial prevalence pattern among COVID-19 patients in Iran, with Gram-negative pathogens predominating. *K. pneumoniae* emerged as the most prevalent organism (36.2%), followed by *A. baumannii* (28.4%). *E. coli* (24.8%), and *P. aeruginosa* (11.7%). Gram-positive bacteria, including *S. aureus* (9.1%) and *coagulase-negative staphylococci* (7.5%), were isolated less frequently. The predominance of Gram-negative bacteria, particularly *K. pneumoniae* and *A. baumannii*, aligns with findings from several global meta-analyses identifying these pathogens as significant causative agents in COVID-19-associated infections [11, 13]. However, the relative frequency of these organisms in our study differs substantially from reports in European settings, where *A. baumannii* prevalence was considerably lower (3% in France, 18.7% in Spain) [19, 20]. This disparity highlights Iran’s distinct antimicrobial resistance ecology. The high prevalence of *K. pneumoniae* (36.2%) observed in our meta-analysis can be attributed primarily to the predominance of ICU patients (63% of cohort) and associated risk factors for healthcare-associated infections [21]. Notably, the high rate of mechanical ventilation in ICU settings created ideal conditions for ventilator-associated pneumonia (VAP), where *K. pneumoniae* is a recognized predominant pathogen due to its environmental persistence and biofilm-forming capabilities [22, 23].

The high prevalence of antibiotic prescriptions in Iranian COVID-19 patients has emerged as a critical driver of antimicrobial resistance, particularly among Gram-negative pathogens. Our meta-analysis revealed alarmingly high rates of AR (71.8%; 95% CI: 63.2–79.1%) and MDR (73.4%; 95% CI: 64.0–82.8%), significantly exceeding global benchmarks. Carbapenem resistance was especially pronounced in *A. baumannii* (91%) and *K. pneumoniae* (74%), far surpassing the 28.7% rate of carbapenem-resistant K. pneumoniae (CRKP) reported in the 2020 WHO GLASS early-warning summary and the 69.2% CRKP observed in ICU settings in high-burden countries [6, 24]. Langford et al.’s meta-analysis of 148 studies documented global antimicrobial resistance of 60.8% (38.6–79.3%) across pathogens, with carbapenem‐resistance rates of 95.2% for *Acinetobacter spp*. and 69.2% for *Klebsiella spp*. These discrepancies reflect differing data sources and timeframes: the 19% figure refers to global CRKP prevalence among invasive isolates, while the 69% estimate encompasses broader MDR definitions in ICU cohorts [6, 24]. Our findings reinforce evidence that low‐ and middle‐income countries face substantially higher AMR burdens than high‐income settings due to systemic stewardship gaps.

Our study reflects the reality that antibiotic prescription rates in Iran during the COVID-19 pandemic were extremely high, with up to 100% of hospitalized patients receiving antibiotics despite low rates of confirmed bacterial co-infection [25, 26]. The situation is further aggravated by the prevalence of extensively drug-resistant (XDR) *A. baumannii* and hypervirulent *K. pneumoniae* ST23/KL1 strains, which are associated with carbapenemase and extended-spectrum β-lactamase (ESBL) production, conferring high-level resistance to multiple antibiotic classes [27–30]. The convergence of high resistance rates and frequent isolation of these organisms in COVID-19 secondary infections underscores the urgent need for robust antimicrobial stewardship and the development of new therapeutic strategies in Iranian healthcare settings.

This study has several limitations that warrant consideration. First, significant heterogeneity in resistance reporting across included studies—stemming from inconsistent MDR definitions and variable antimicrobial susceptibility testing protocols—constrained our ability to generate precise pooled estimates of AR and MDR. Only three bacterial species (*Klebsiella pneumoniae*,* Acinetobacter baumannii*, and *Pseudomonas aeruginosa*) had sufficient MDR data for meta-analysis, limiting generalizability to other pathogens. Second, while most studies focused exclusively on COVID-19 patients, some did not confirm SARS-CoV-2 infection via PCR, potentially introducing selection bias; we broadly defined our cohort as patients treated during the COVID-19 era, but this may have included non-COVID-19 cases with distinct AMR profiles. Third, the predominance of ICU-derived data (63% of patients) and regional clustering (33% of studies from Tehran) may skew results toward hospital-acquired, carbapenem-resistant Gram-negative pathogens, underrepresenting community-acquired resistance trends. Fourth, limiting our search to English-language publications may have excluded relevant Persian-language studies, introducing language bias and restricting the comprehensiveness of our evidence synthesis. Finally, the very high heterogeneity observed in our meta-analyses (I² >89% for most outcomes) further limits confidence in pooled point estimates. This heterogeneity reflects clinical diversity (varying patient ages, comorbidities, COVID-19 severity, and specimen types), methodological variation (different study designs, sampling methods, diagnostic criteria, and testing protocols), statistical differences (wide variation in effect sizes and confidence intervals across facilities and regions), and temporal shifts (studies spanning January 2020 to March 2025, capturing evolving resistance patterns and infection control practices). Although we used random-effects models to account for heterogeneity, readers should interpret pooled estimates with caution and focus on the observed range of resistance patterns rather than precise point values. Despite these limitations, our systematic assessment provides critical insights into Iran’s antimicrobial resistance landscape during the COVID-19 pandemic.

## Conclusion

Overall, this systematic review demonstrates that AR and MDR rates among COVID-19 patients in Iran were alarmingly high, particularly for Gram-negative pathogens. Carbapenem resistance was most pronounced in *A. baumannii*, *K. pneumoniae*, and *P. aeruginosa*. Resistance to third generation cephalosporins was also widespread, especially in *K. pneumoniae* and *E. coli*. These findings highlight the urgent need for enhanced antimicrobial stewardship, standardized surveillance, and targeted interventions to address the escalating threat of AR in Iranian healthcare settings during and beyond the COVID-19 pandemic. These baseline data can guide policymakers in Iran to monitor trends and evaluate the impact of stewardship interventions in the post-pandemic era.

## Supplementary Information

Supplementary Material 1.

## Data Availability

No datasets were generated or analysed during the current study.
